# Properties of Novel Non-Silicon Materials for Photovoltaic Applications: A First-Principle Insight

**DOI:** 10.3390/ma11102006

**Published:** 2018-10-17

**Authors:** Murugesan Rasukkannu, Dhayalan Velauthapillai, Federico Bianchini, Ponniah Vajeeston

**Affiliations:** 1 Department of Computing, Mathematics and Physics, Western Norway University of Applied Sciences, Inndalsveien 28, 5063 Bergen, Norway; vdh@hvl.no; 2 Department of Chemistry, Center for Materials Science and Nanotechnology, University of Oslo, Box 1033, Blindern, N-0315 Oslo, Norway; federico.bianchini@smn.uio.no (F.B.); ponniah.vajeeston@kjemi.uio.no (P.V.)

**Keywords:** HSE06, non-silicon, non-conventional solar cells, PV materials, hybrid density function, BSE

## Abstract

Due to the low absorption coefficients of crystalline silicon-based solar cells, researchers have focused on non-silicon semiconductors with direct band gaps for the development of novel photovoltaic devices. In this study, we use density functional theory to model the electronic structure of a large database of candidates to identify materials with ideal properties for photovoltaic applications. The first screening is operated at the GGA level to select only materials with a sufficiently small direct band gap. We extracted twenty-seven candidates from an initial population of thousands, exhibiting GGA band gap in the range 0.5–1 eV. More accurate calculations using a hybrid functional were performed on this subset. Based on this, we present a detailed first-principle investigation of the four optimal compounds, namely, TlBiS_2_, Ba_3_BiN, Ag_2_BaS_2_, and ZrSO. The direct band gap of these materials is between 1.1 and 2.26 eV. In the visible region, the absorption peaks that appear in the optical spectra for these compounds indicate high absorption intensity. Furthermore, we have investigated the structural and mechanical stability of these compounds and calculated electron effective masses. Based on in-depth analysis, we have identified TlBiS_2_, Ba_3_BiN, Ag_2_BaS_2_, and ZrSO as very promising candidates for photovoltaic applications.

## 1. Introduction

The solar energy reaching the earth amounts to approximately ten thousand times the primary energy usage by the world population. Solar photovoltaic cells are among the most important technologies for clean energy production. It is predicted that in future, the power from solar photovoltaic modules will reach the terawatt level [1]. Photovoltaic (PV) technology is currently dominated by silicon solar cells. If we look at the worldwide scenario, more than 80% of the installed PV modules are mainly mono or multi-crystalline silicon based [1]. However, researchers are making considerable efforts to develop solar cells based on alternative materials because silicon is an indirect band gap material with a low absorption coefficient. Novel materials considered for PV applications include copper zinc tin sulfide (CZTS), ternary, binary, and multinary compounds with a direct band gap, enabling high absorption properties. High photon conversion efficiency and low production cost are the other desired features of these alternative materials. There is also considerable interest in the research community to find ways to develop solar cells that have efficiencies greater than the Shockley-Queisser limit of 32% [2].

The development of non-silicon materials is a very active field, and several significant signs of progress have been made recently [1]. In the future, non-silicon materials will most likely be produced using thin film technologies, with a resulting device thickness in the order of 2 μm. Despite the crucial role played by these compounds for the next generation of energy materials, the current knowledge of the optical and electronic properties of these compounds is inadequate. Non-silicon materials such as organic semiconductors may become the main candidates for future photovoltaic devices, even though they have low stability [3]. To be a promising solar cell material, a semiconductor preferably have a direct band gap with an appropriate band gap value resulting in efficient absorption of the solar spectrum.

Furthermore, it can be used in a junction formation, which is appropriate for guiding the electrical processes involved in energy conversion [4]. A variety of basic materials, GaAs, InP, CdTe, CuInSe_2_ to name a few, and large band gap materials such as ZnO, CdS, ZnCdS used as window layers in creating heterojunctions have been studied extensively [4]. Reducing production cost is one of the priorities in selecting materials for PV technologies. Compared to mono-crystalline Silicon solar cells, the production cost for poly-crystalline Silicon solar cells is lower, but the efficiency is lower [5]. According to Mitchell [6], materials that require only a few micrometers of thickness to absorb the solar spectrum and photo-carriers effectively are created close to the electrical junction. One way to minimize material usage is to choose direct band gap materials over indirect band gap materials, since direct band gap solar cells could be made substantially thinner [7]. Due to the low absorption, polycrystalline silicon solar cell structures must have a thickness in the range of 200 μm, which makes the overall cost higher. We have earlier reported several non-silicon intermediate band gap materials that can be used for solar cell applications [8,9]. Our main aim in this study is to propose non-silicon based direct band gap materials with highly efficient photoelectric properties such that material costs become lower.

As one of the most effective and accurate computational methods for modelling atomistic systems, density functional theory (DFT) has been widely applied in this work to extensively analyze the electronic band structure of thousand non-silicon based materials in order to identify candidates that have a band gap between 0.5 eV and 1.1 eV. The band structure calculation for these presented materials is based on the generalized gradient approximation (GGA) that underestimates the value of the band gap. However, this technique is efficient and time-effective in terms of computational resources, and it can be used for an initial screening of a large number of compounds. The initial structural parameters of thousand compounds were directly taken from the ICSD database [10], and then the GGA band gap for thousands of non-silicon compounds were calculated in our DFTB database [11]. These are multinary compounds including conductors, semiconductors, and insulators. Among these thousand non-silicon compounds, we considered twenty-seven of them with GGA band gap values in the range of 0.5–1.1 eV (Table S1 of Supplementary Materials). Among these twenty-seven compounds, we identified fourteen compounds as direct band gap semiconductors and thirteen as indirect band gap semiconductors. We carried out a study on both the electronic and optical properties of twenty-seven semiconductors (both direct and indirect). Our study of the optical properties of semiconductor materials showed that four direct band gaps among the twenty-seven materials had higher absorption coefficients in the visible region. Due to the space constraint, the optical properties of all the twenty-seven semiconductors are not presented in the supporting information.

We carry out a comprehensive study on these four materials, namely TlBiS_2_, Ba_3_BiN, Ag_2_BaS_2_, and ZrSO, and report accurate electronic structure results for these four compounds by employing a more accurate calculation based on the screened–exchange hybrid density functional Heyd-Scuseria-Ernzerhof (HSE06). HSE06 helps to identify the contributions of individual elements to the electronic structure of the compounds. We also study the structural and mechanical stability and the optical properties of these four materials to verify the applicability of these four materials for photovoltaic applications. In a recently published article, we focused on TlBiS_2_, and have presented electronic band structure and optical spectra based on spin-orbit coupling (SOC) [12].

## 2. Computational Details

We employed DFT analysis using Vienna ab initio simulation package (VASP, 5.4.1. Feb. 16) with the projected augmented plane-wave (PAW) approach [13] to study the electronic structures of TlBiS_2_, Ba_3_BiN, Ag_2_BaS_2_, and ZrSO. The Perdew–Burke–Ernzerhof (PBE) functional is used to treat exchange and correlation within the GGA approach [14]. To obtain an improved description of the interaction between oxygen and the transition metal atoms, we used the Hubbard parameter correction (DFT+U), following the rotationally invariant form [15,16,17]. The full details about the computed *U* and *J* values are presented in the DFTBD database website [11]. These DFT+U calculations are used for the structural optimisation of the considered compounds, as the relaxation using HSE06 is time-consuming and has no significant effect on the structural properties. Ground-state geometries are calculated using the conjugate-gradient algorithm with a force convergence threshold of 10^−3^ eV Å^−1^, and minimizing the stress tensor and the Hellman-Feynman forces. In order to achieve better and detailed band structures, we used the hybrid functional of Heyd–Scuseria–Ernzerhof (HSE06). For the standard HSE06 functional, the screened parameter was assigned to 0.2 A^−1^, and the screened Hartree-Fock (HF) exchange was set to 30% mixing with the PBE exchange functional [18]. The cut-off energy for the plane-wave basis expansion was set to 600 eV, and for Brillouin zone integration we used a 6 × 6 × 6 Г-centered Monkhorst-pack **k**-point mesh. In both calculations (i.e., PBE and HSE06), this setting is used.

Solving the Casida’s equation is one of the best approaches for determining the dielectric function [19]. We have summed the contributions over a number of 8 × 8 × 8 **k**-points grids, shifted with respect to each other to reproduce16 × 16 × 16 Г-centered **k**-points mesh, using a plane-wave cut-off of 410 eV for both GW and Bethe-Salpeter equation (BSE). To get a more accurate peak position and intensities in optical spectra, the optical calculation counts the contribution from 200 electronic bands. For all of these computations, the initial structural parameters were taken directly from the ICSD database [10]. The information about the high symmetric points of the *k*-vector in the Brillouin zone was taken from the Bilbao Crystallographic Server [20,21,22].

A frozen phonon calculation was performed on suitable supercells of the relaxed structures, generated using the phonopy program (1.9.2). This software is also used to obtain the phonon dispersion curve and the phonon density of states from the converged calculations [23,24]. The atomic displacement of 0.0075 is used, with symmetry considerations to obtain the force constants for the phonon calculations. The displacements in opposite directions along all the axes were incorporated into the calculations to improve the precision. The force calculations were performed using the VASP code (DFT+U level), and the resulting data were imported into PHONOPY. The dynamical matrices were derived from the force constants, and phonon DOS curves were computed using the Monkhorst-Pack scheme [25].

## 3. Results and Discussion

### 3.1. Structural Properties

**Figure 1:**
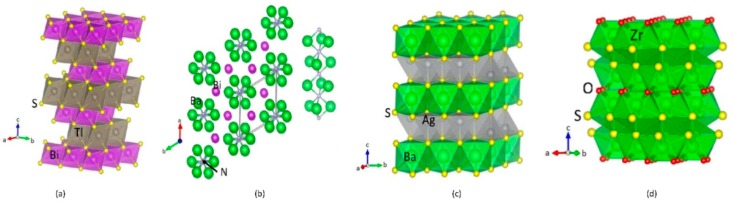
Crystal structures for (**a**) TlBiS_2_; (**b**) Ba_3_BiN; (**c**) Ag_2_BaS_2_; (**d**) Tetragonal-ZrSO. The illustration shows the legends for the different type of atoms.

**TlBiS_2_:** TlBiS_2_ has rhombohedral structures with space group D5 3d (R-3m, Space group No. 166), similar to Bi_2_Te_3_. There are four atoms, namely, 1-Tl, 1-Bi, and 2-S positioned in layers normal to the three-fold axis in the arrangement Tl-S-Bi-S, as shown in Figure 1a. Each Tl/Bi layer is placed between two S layers, which indicates a strong interlayer coupling so that the crystal structure is substantially three-dimensional. Tl, Bi, and S are placed at the (0, 0, 0), (0.5, 0.5, 0.5), and (±u, ±u, ±u) sites, respectively. This structure has inversion symmetry where both Bi and Tl represent inversion centers. The basic TlBiS_2_ structure is a simple NaCl-type lattice. It is similar to *ABQ*_2_-type compounds (*A*, *B* and *Q* are monovalent atom, trivalent atom and chalcogen respectively). The TlBiS_2_ structure is rhombohedral along the cubic [111] direction and matching to the *c* axis of the primitive hexagonal arrangement. The sum of the ionic radii for a coordination number (CN) of 6 is 2.87 Å for Bi^3+^/S^2−^ and 3.34 Å for Tl^+^/S^2−^ [26]. The experimentally-determined and theoretically-derived bond length matches well for Bi-S, but for the Tl-S distance the value is about 5.3% smaller. Our calculated lattice parameters and the positional parameters all fitted well with the experimental findings (see Table S2) [26].

**Ba_3_BiN:** (Ba_3_N)Bi crystallizes in a hexagonal anti-perovskite variant of the BaNiO_3_ structure type (*P*63/*mmc*, space group No. 194, *Z* = 2). This phase consists of Ba_6_N octahedral units sharing faces formed with three Ba ions according to a rod-like structure along [001] (see Figure 1b). The calculated Ba-N distance is 2.677 Å which compares well with those in sub-nitrides with nitrogen species in octahedral coordination [27,28]. Hexagonal perovskite crystal structures can only be expected for compounds containing alkaline-earth metal species with large radii. Hence, the resulting distance *d* (N-N) and the Coulomb repulsion between N^3−^ in face-sharing octahedra has to be formed. The resulting distance N-N is 3.3218 Å is sufficiently large.

**Ag_2_BaS_2_:** Ag_2_BaS_2_ crystallises in the trigonal CaA1_2_Si_2_-type structure, a = 4.386 (1) A, *c* = 7.194 (2) A, space group P3m1, *Z* = 1, where S and Ag atoms are arranged in the chemically ordered double-corrugated hexagonal layers and Ca atoms are intercalated between them [29], as shown in Figure 1c. These layers can, in turn, be described as being made up from two stacked AgS layers, with each layer being a two-dimensional infinite net of chair-like six-membered rings. Every atom in the Ag_2_S_2_ layer is four-coordinate, but the coordination environment is very different for Ag and S. Each Ag is surrounded by four S atoms, forming a distorted tetrahedron. The S is also four-coordinate in Ag, but the environment is most unusual, a flipped tetrahedron or umbrella shape.

**ZrSO:** ZrSO crystalizes in cubic (P2_1_3, space group No. 198) and tetragonal (P4/nmm, space group No. 129) form [30,31]. Tetragonal ZrSO crystallises in the PbFCl-type structure. The form of this phase has not yet been synthesized [31]. All preparations techniques proved that the tetragonal phase was always accompanied by considerable proportions of cubic ZrSO, and in some cases, even by ZrO_2_. It seemed likely, therefore, that the tetragonal phase also contains oxygen [31]. According to our theoretical energy volume curve (see Figure S1), the cubic form is more stable than the tetragonal form and the energy difference between these two structures is very small (36 meV/f.u.). Moreover, it is clear from Figure S1 that the energy minima for these two structures are well separated, and the energy well is deep enough to stabilize the individual phases. We can thus conclude that this phase can be experimentally stabilized using a high-pressure technique.

There are four S and three O atoms surround each Zr atom in the cubic phase. The S atoms form a stretched tetrahedron with one Zr-S separation of 2.61 Å and three of 2.63 Å. The O atoms form an equilateral triangle centered at the Zr site, in such a way that there is an O atom in each of the stretched faces of the S atoms tetrahedron. At a distance of 2.13 Å from each oxygen atom, the Zr atom is slightly out of the plane of the oxygen triangle. The configuration may be regarded as a distorted octahedron consisting of three S atoms and three O atoms, with an extra sulfur atom above the center of the face of the octahedron driven by the oxygen atoms. From this point of view, the co-ordination of zirconium is quite similar to that observed in K_3_ZrF_7_. The separations of S-S and S-O are 3.59 and 2.96 Å, respectively. These separations are shorter than the sum of the radii (3.68 Å for S-S and 3–24 Å for S-O) owing to the sharing of edges between the coordination polyhedra [30].

### 3.2. Electronic Properties

**Figure 2:**
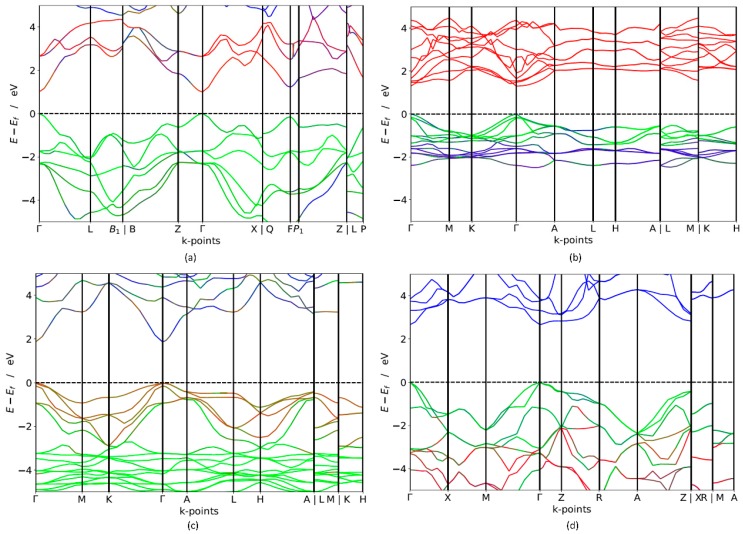
Calculated electronic band structure (using HSE06) of (**a**) TlBiS_2_(Colour code: red line—Bi, green line—S, blue—Tl), (**b**) Ba_3_BiN (Colour code: red line—Ba, green line—Bi, blue—N), (**c**) Ag_2_BaS_2_ (Colour code: red line—S, green line—Ag, blue—Ba) and (**d**) ZrSO (Colour code: red line—O, green line—S, blue—Zr). The Fermi level is set to zero.

In order to investigate the potential applicability of non-silicon semiconductors as a light-harvesting medium, the band gap of these materials is a crucial factor that needs to be further explored. Both short-circuit current and open-circuit voltage is regulated by the band gap of the photoactive semiconductors. Broader band gap leads to higher open-circuit voltage but fewer excited electrons, which results in lower short-circuit current. Narrower band gap leads to low open circuit voltage but more excited electrons, which result in larger short-circuit current. The ideal solar cell is theoretically shown to have a maximum of 32% efficiency with an optimal band gap E_g_ = 1.4 eV is [2]. In real cells, the solar spectrum is a broad energy spectrum and it does not match the band gap well where thermalisation loss occurs, which eventually results in efficiencies below the detailed-balance limit [2]. Band gap calculation using electronic band structures gives a promising opportunity to identify suitable PV materials. The calculated band structure of trigonal-TlBiS_2_, hexagonal-Ba_3_BiN, trigonal-Ag_2_BaS_2_ and tetragonal-ZrSO crystals along a high-symmetry path in the first Brillouin zone are presented in Figure 2. For electronic structure calculations, we employ hybrid functional (HSE06) and estimate the band gap values for these materials. The results of electronic structure calculations, listed in Table 1, span the in the range from 1.10 to 2.6 eV. The four compounds exhibit a direct band gap at the Г **k**-point, as shown in Figure 2.

**Figure 3:**
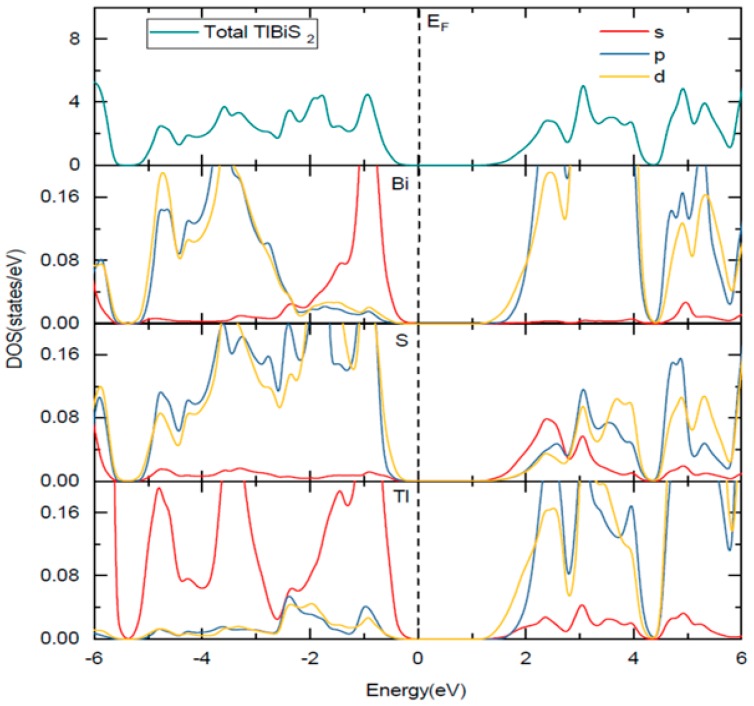
Total and site projected density of states of TlBiS_2_. The Fermi level is set to zero. The Fermi level is set to zero and marked by a vertical dotted line.

The HSE06 band structure of TlBiS_2_ is shown in Figure 2a. Both the valence band maximum (VBM) and the conduction band minimum (CBM) are well placed at the Г **k**-point. This clearly shows that TlBiS_2_ is a direct band gap semiconductor with valence bands derived from Bi-s, S-p, and Tl-s states, and conduction bands derived from S-s, Bi-p, and Bi-d states (Figure 3). The HSE06 band gap is 1.1 eV for TlBiS_2_, which is nearly equal to that of Silicon. Bahadur Singh et al., showed that TlBiS_2_ has GGA band gap of 0.64 eV with direct band gap type at Г **k**-point [32]. It is important to note that we employed a more accurate HSE06 method compared to the GGA calculation method used by Bahadur Singh et al. [32] and the difference is approximately 0.46 eV. This is as expected because it is well known that calculations using GGA underestimate the band gap value, while the HSE06 screened hybrid functional is very successful in precisely calculating the band gap value. Our calculation shows that we have a band gap of 1.42 eV at F **k**-point TlBiS_2_ as shown in Figure 2a. This shows that TlBiS_2_ well suited for PV applications as optimal band gap for the best performance is 1.4 eV as mentioned earlier [12].

In the case of Ba_3_BiN, the VBM and CBM are located at Г **k**-point. Thus, the calculated HSE06 band structure in Figure 2b shows that Ba_3_BiN is a direct band gap semiconductor with a band gap of 1.29 eV. From the Figure S3 of Supplementary Materials, the valence band derived from Bi-p and hybridized Ni-d states and conduction bands are mainly composed of Bi-s and Ba-s states. According to Imran Ullah et al., Ba_3_BiN is a direct band gap semiconductor with a band gap of 0.64 eV at Г **k**-point [33]. The comparison between the present results using HSE06 with the previous results using GGA [33] reveals that the band gap of Ba_3_BiN is previously underestimated by 0.79 eV. To the best of our knowledge, no HSE06 or experimental studies have been previously reported on Ba_3_BiN. For Ag_2_BaS_2_, the calculated HSE06 band structure in Figure 2c shows that the VBM and CBM are located at the Г **k**-point. Thus, Ag_2_BaS_2_ is a direct band gap semiconductor with a band gap of 1.95 eV. Note that our direct band gap value of 1.95 eV calculated with HSE06 closely matches the previous HSE06 band gap value of 2.01 eV [34] calculated by Aditi Krishnapriyan et al. To the best of our knowledge, no experimental study has been previously reported on Ag_2_BaS_2_. From Figure 2b and the Figure S4 of Supplementary Materials, the valence band maximum is derived from S-p states and conduction band derived from Ag-s states. In the case of ZrSO, both the VBM and the CBM are located at Г. Thus, ZrSO is a direct band gap semiconductor with valence bands derived from S-p states, and conduction bands derived from O-s states (Figure S5). The calculated HSE06 band gap between VBM and CBM is 2.60 eV. To the best of our knowledge, no HSE06 and experimental study have previously reported on ZrSO.

### 3.3. Effective Mass Calculation

We calculate the conductivity effective masses for all four materials; calculated values are listed in Table 2. To study of energy levels in solar devices, calculations of the effective mass (EM) play a crucial role. The conductivity effective masses of electrons and holes deal with the mobility, electrical resistivity, and free-carrier optical response in the semiconductor material used in PV applications. For EM calculation, we have used a finite difference method as implemented in the effective mass calculator (EMC) [35]. For TlBiS_2_, Ba_3_BiN, and Ag_2_BaS_2_, the EM of holes was found to be heavier than the EM of electrons. This result can be ascribed to the fact that the VBM is less dispersed than the CBM. Prominent PV materials Silicon (Si) and gallium arsenide (GaAs) have EMs of 0.26 m_e_ and 0.12 m_e_ [36] respectively for electrons. The calculated EM of electrons are 0.154 m_e_, 0.092 m_e_, and 0.149 m_e_ for TlBiS_2_, Ba_3_BiN, and Ag_2_BaS_2_ respectively. Compared to Si, for TlBiS_2_, Ba_3_BiN, and Ag_2_BaS_2_, the EM of the electron is lower. Hence, the electron mobility in these three compounds is better than that of silicon. However, in the case of ZrSO, the EM of the hole is lighter than the EM of electrons; this is due to CBM being less dispersed than VBM in ZrSO.

### 3.4. Lattice Dynamic Stability

**Figure 4:**
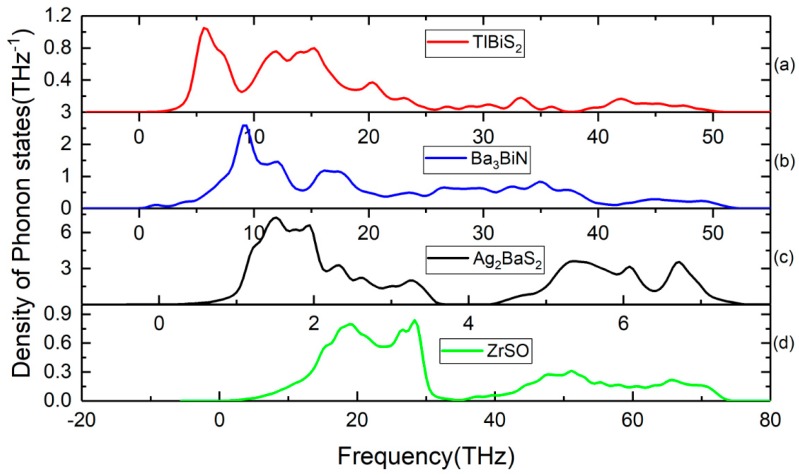
Calculated total phonon density of states for (**a**) TlBiS_2_, (**b**) Ba_3_BiN, (**c**) Ag_2_BaS_2_, and (**d**) ZrSO phases.

**Figure 5:**
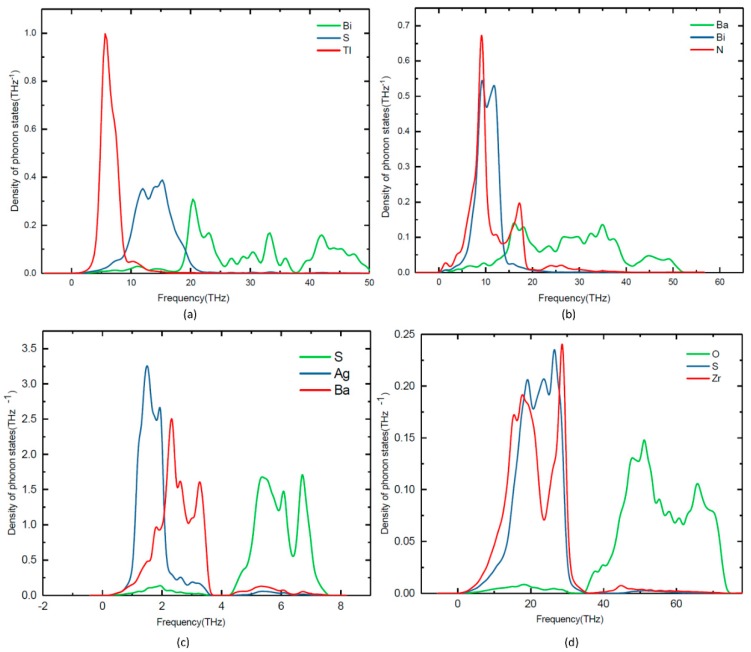
Calculated site projected phonon density of states for (**a**) TlBiS_2_, (**b**) Ba_3_BiN, (**c**) Ag_2_BaS_2_, (**d**) ZrSO.

Lattice dynamic calculations have also been performed on TlBiS_2_, Ba_3_BiN, Ag_2_BaS_2_, and ZrSO under ambient conditions. To validate the dynamical stability of these compounds, the total phonon density of states is calculated at the equilibrium volume. In Figure 4, we displayed their total phonon density of states. No imaginary frequencies were observed, revealing that TlBiS_2_, Ba_3_BiN, Ag_2_BaS_2_, and ZrSO are dynamically stable. We present the site projected phonon density of states for TlBiS_2_, Ba_3_BiN, Ag_2_BaS_2_, and ZrSO in Figure 5. The vibrational modes spread over the 2 to 50 THz range in the case of TlBiS_2_ phase. In the low frequencies region, Tl is dominant over Bi and S. The lattice vibrational modes for the Tl, S, and Bi are present between 3–10, 5–21, and 18–50 THz, respectively. In the case of Ba_3_BiN, the vibrational modes spread over 0 to 52 THz. The lattice vibrational modes for N, Bi, and Ba are present between 0–19, 2–20 and 3–51 THz, respectively. For Ag_2_BaS_2_, the vibrational modes spread over 0.3 to 7.5 THz. The Ag-S and Ba-S stretching modes are present between 1–3 THz. Other Ag-Ba, S-Ag, and S-Ba stretching modes are present between 5–6.5, 5–6.5 and 4.3–7 THz, respectively. The lattice vibrational modes for Ag, Ba, and S are present between 0.3–3.6, 0.3–3.6, and 4.3–7.5 THz, respectively.

In the case of ZrSO, the vibrational modes spread over 2 to 74 THz. The Zr-O and S-O stretching modes are present between 5–30 THz. Other O-Zr and O-S stretching modes are present at 40–65 and 47–60 THz, respectively. The lattice vibrational modes for Zr, S, and O are present between 2–35, 2–35, and 35–74 THz, respectively. The calculated zero-point energy (ZPE) for the TlBiS_2_, Ba_3_BiN, Ag_2_BaS_2_, and ZrSO phases varies from 0.10 to 1.3 eV/f.u. (see Table 3), and the following ZPE sequence are: Ag_2_BaS_2_ < TlBiS_2_ < ZrSO < Ba_3_BiN.

**Figure 6:**
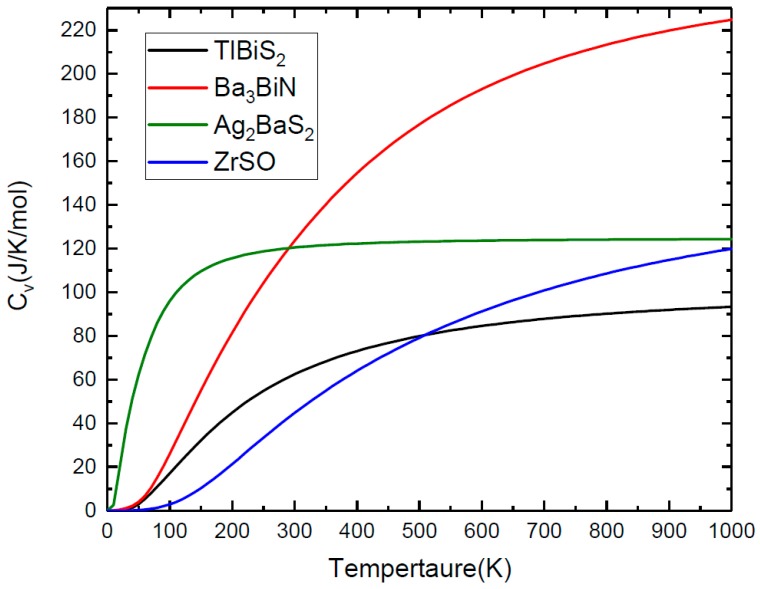
The heat capacity at constant volume as a function of temperature from zero up to 1000 K.

In addition to the dynamic stability, we employ the vibrational density of states to compute the specific heat capacity (C_v_) of TlBiS_2_, Ba_3_BiN, Ag_2_BaS_2_, and ZrSO at constant volume and pressure. The C_v_ as a function of temperature presented in Figure 6 in the temperature range from 0 K to 1000 K. For TlBiS_2_, the specific heat capacity increases rapidly below 500 K. The value of C_v_ is almost constant at 90 J/K/mol for above 500 K. In the case of Ba_3_BiN, the C_v_ increases rapidly up to 1000 K. The specific heat capacity increases rapidly below 100 K for Ag_2_BaS_2_. The C_v_ is almost constant at 125 J/K/mol for above 100 K. For ZrSO, the C_v_ increases from 100 K to 1000 K. The following C_v_ sequence are: TlBiS_2_ < ZrSO < Ag_2_BaS_2_ < Ba_3_BiN.

### 3.5. Mechanical Stability

The mechanical stability of a system is an essential condition to validate the existence of a compound in a given crystalline structure. The elastic constants are typically used to describe the mechanical properties of a system and to estimate its hardness. To validate the mechanical stability of TlBiS_2_, Ba_3_BiN, Ag_2_BaS_2_, and ZrSO, we calculated the single-crystal elastic constant tensor using the finite strain technique. The elastic constants describe the ability of materials to deform, or conversely, the stress required to maintain a given deformation. Both stresses and strains have three tensile and three shear components. The linear elastic constants form a 6 × 6 symmetric matrices, with 27 independent components, so that (*s_i_* only) *s_ij_* = C*_ij_* *ε_j_* (*s_i_* is stress tensor, C*_ij_* is elastic constant matrix, *ε_j_* (*j* = 1, 6 in Voigt index) is the strain tensor, and *i* = 1, …, 6) for small stresses and strains [37].

The stiffness of a crystal against an externally applied strain can be determined from its elastic constants. Any symmetry present in the structure may make some of these components equal, while others may be fixed to zero. The calculated elastic constants of four non-silicon materials listed in Table 4. The elastic constant C_44_ is a crucial parameter, indirectly describing the indentation hardness of the materials. As shown in Table 4, all the examined compounds have a small C_44_ value, indicating these materials possess a relatively weak shear strength.

For trigonal structures, the mechanical stability criteria at zero pressure are as follows [38]:

C_11_ > |C_12_|, C_11_ > 0, C_33_ > 0, C_44_ > 0 (1)

[(C_11_ + C_12_) C_33_ − 2C^2^_13_] > 0 (2)

[(C_11_ − C_12_) C_44_ − 2C^2^_14_] > 0 (3)

In this study, trigonal TlBiS_2_ and Ag_2_BaS_2_ have six independent elastic constants. All the three mechanical stability conditions given in Equations (1)–(3) are satisfied for the TlBiS_2_ and Ag_2_BaS_2_ phases. Hence, this indicates that these two trigonal phase materials are mechanically stable.

For the hexagonal system, the Born stability criteria are [38]:

C_44_ > 0 (4)

C_11_ − |C_12_| > 0 (5)

[(C_11_ + C_12_) C_33_ − 2C^2^_13_] > 0 (6)

The hexagonal-Ba_3_BiN has five independent elastic constants. All three conditions for the mechanical stability given in equations 4 to 6 are satisfied for this structure, and this finding indicates that hexagonal-Ba_3_BiN phases are mechanically stable.

The mechanical stability criteria for the tetragonal phase are given by [38]:

C_11_ − C_12_ (7)

2(C_11_ + C_12_) + C_33_ + 4C_13_ (8)

C_44_ > 0, C_66_ > 0 (9)

C_11_ + C_12_ − 2C_13_ > 0 (10)

ZrSO has a tetragonal structure, and thus, six independent elastic constants. All three conditions for mechanical stability given in Equations (7)–(10) are satisfied for this structure. Hence, the tetragonal-ZrSO phase is mechanically stable at ambient conditions. Equations (1)–(9) and Table 4 validated the mechanical stability criteria for the crystal under ambient conditions. This outcome is consistent with the phonon calculations presented in Section 3.4.

From the calculated elastic constants, the bulk (B_v_, B_R_) and shear moduli (G_v_, G_R_) are calculated from Voigt–Reuss–Hill approximations [39,40]. The bulk and shear moduli contain information related to the hardness of the material under various types of deformation. Generally, very hard materials hold very large bulk and shear moduli to support the volume decrease and to restrict deformation, respectively [41]. From Table 4, it can be identified that the listed TlBiS_2_, Ba_3_BiN, and Ag_2_BaS_2_ phases have a smaller bulk modulus than ZrSO (181.403 GPa). This indicates that ZrSO is more difficult to compress than the other three materials. Among these compounds, the bulk modulus sequence is: ZrSO > Ag_2_BaS_2_ > TlBiS_2_ > Ba_3_BiN. As we know, the shear modulus is more closely-connected to hardness than the bulk modulus. From Table 4, the shear modulus of ZrSO is higher than the other three compounds. Hence, the hardness of the tetragonal-ZrSO phase is higher than trigonal-TlBiS_2_, hexagonal-Ba_3_BiN, and trigonal-Ag_2_BaS_2_. Among these compounds, the shear modulus trend is ZrSO > TlBiS_2_ > Ag_2_BaS_2_ > Ba_3_BiN. Seemingly, the bulk and shear moduli of Ba_3_BiN are smaller than other compounds. Thus, Ba_3_BiN is easy to compress and is the softest of the examined materials.

The parameter G/B can be introduced, in which G indicates the shear modulus and B the bulk modulus. The low/high of G/B value is connected with the ductility or brittleness of the materials. The critical G/B value that separates the ductile and brittle materials is 0.5 [41]. If the G/B value of materials is smaller than 0.5, then those materials are ductile; otherwise they are brittle. From Table 4, the calculated G/B values of all four materials are greater than 0.5, indicating that these materials are ductile. Next, the value of Poisson’s ratio is indicative of the degree of directionality of the covalent bonding. Among these compounds, the small Poisson’s ratio (0.13) for hexagonal-Ba_3_BiN indicates a high degree of covalent bonding. All these phases present a very scattered Young’s (varying from 29 to 339 GPa). The compressibility value of these phases suggests that these compounds, with the exception of ZrSO, are very soft materials. The compressibility sequence is ZrSO < Ag_2_BaS_2_ < TlBiS_2_ < Ba_3_BiN.

### 3.6. Optical Properties

The optical behavior of a compound has a major impact on its properties for photovoltaic applications. Optical dielectric function ε(ω) = ε_1_(ω) + ιε_2_(ω) is the fundamental quantity that describes the optical properties of a material. It describes the response of a material to a radiated electromagnetic field and the propagation of the field inside the material. The dielectric function is dependent on the frequency of electromagnetic field, and it is connected to the interaction between photons and electrons. The absorption coefficient of the material is dependent on the imaginary part, ε_2_(ω), and it can be derived from the inter-band optical transitions by summing over the unoccupied states, using the equation [42,43],

(11) $$\begin{array}{c} \varepsilon_{2}^{\left(\alpha \beta \right)}\left(\omega \right)=\frac{4\pi^{2}e^{2}}{Ω}\;\underset{q\to 0}{\lim}\;\sum_{k,v,c}2w_{k}\delta (\epsilon_{ck}-\epsilon_{vk}-\omega )\\ \;\;\;\times \langle u_{ck+e_{\alpha }q}\left|u_{vk}\rangle \langle u_{ck+e_{\beta }q}\right|u_{vk}\rangle^{*} \end{array}$$

where the indices *α*, *β* are the Cartesian components, Ω is the volume of the primitive cell, *q* denotes the Bloch vector of the incident wave, *c* and *v* are the conduction and valence band states respectively, *k* is the Bloch wave vector, *w_k_* denotes the **k**-point weight, δ is a Dirac delta function, *u_ck_* is the cell periodic part of the orbital at **k**-point k, $\epsilon_{ck}$ refers to the energy of the conduction band, and $\epsilon_{vk}$ refers to the energy of the valence band. The real part ε_1_(ω) of the dielectric function can be derived from ε_2_(ω) using the Kramer-Kronig relationship [42,43]

(12) $$\varepsilon_{1}^{\left(\alpha \beta \right)}=1+\frac{2}{\pi }P\int_{0}^{\infty }\frac{\varepsilon_{\alpha \beta }^{2}(\omega^{\prime })\omega^{\prime }}{\omega^{\prime 2}-\omega^{2}+i\eta }d\omega^{\prime }$$

where P indicates the principal value, η is the complex shift. All the frequency dependent linear optical properties, such as the absorption coefficients α(ω), can be calculated from ε_1_(ω) and ε_2_(ω) [42,43].

(13) $$\alpha \left(\omega \right)=\frac{\sqrt{2\omega }}{c}\left[{\left(\varepsilon_{1}^{2}\left(\omega \right)+\varepsilon_{2}^{2}\left(\omega \right)\right)}^{\frac{1}{2}}-\varepsilon_{1}\left(\omega \right)\right]$$

**Figure 7:**
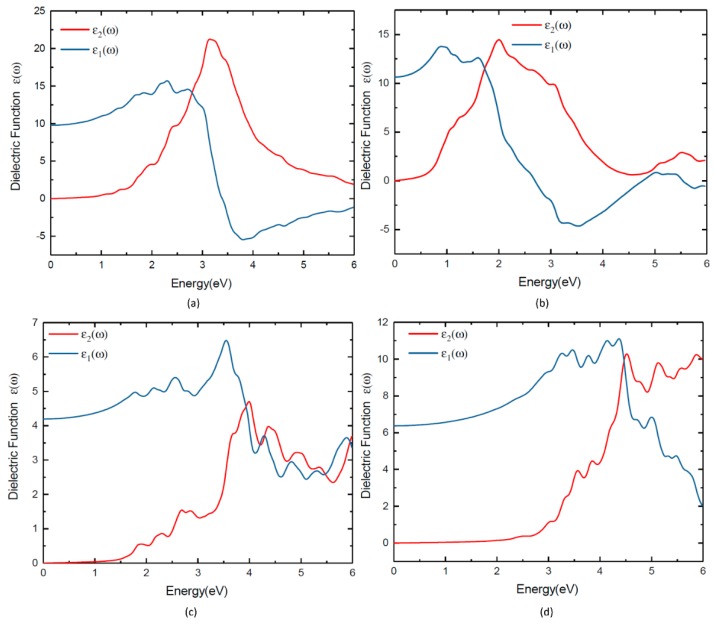
Dielectric function of (**a**) TlBiS_2_, (**b**) Ba_3_BiN, (**c**) Ag_2_BaS_2_ and (**d**) ZrSO.

**Figure 8:**
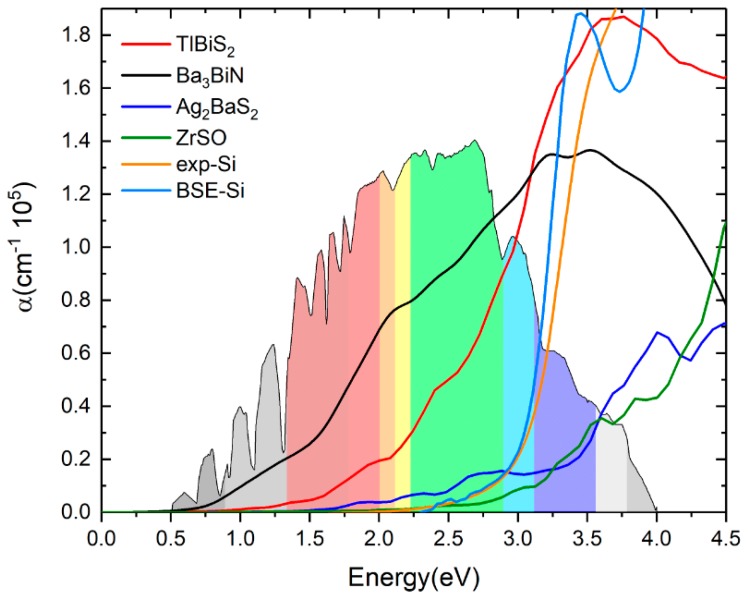
Optical absorption coefficients of TlBiS_2_, Ba_3_BiN, Ag_2_BaS_2_ and ZrSO. For comparison, we plotted the silicon optical absorption coefficient (color code: orange—experimental absorption coefficient of Si, navy blue—BSE calculation of Si).

Experimental absorption spectra are in agreement with the inclusion of excitonic effects treated within the Bethe-Salpeter equation (BSE) in general [44,45,46]. By averaging multiple grids using BSE, the calculated the dielectric function of these four materials can be further improved. The calculated ε_2_(ω) of the dielectric function and the absorption coefficients of TlBiS_2_, Ba_3_BiN, Ag_2_BaS_2_, and ZrSO are presented in Figure 7 and Figure 8, respectively. From the directional dependency of ε_1_(ω) and ε_2_(ω), trigonal-TlBiS_2_ is highly isotropic, whereas hexagonal-Ba_3_BiN, trigonal-Ag_2_BaS_2_, and tetragonal-ZrSO are less anisotropic. We present the average of the real and imaginary parts of the dielectric function for the four examined compounds.

In Figure 7, we plotted both real and imaginary part of the dielectric function of (a) TlBiS_2_, (b) Ba_3_BiN, (c) Ag_2_BaS_2_, and (d) ZrSO is plotted against the photon energy. The optical absorption coefficients of all these materials were calculated using BSE and plotted in Figure 8. For a comparison, we have also plotted both the experimentally-verified [47] and the BSE-calculated [19] values for the optical absorption coefficient of silicon in the same Figure 8. Absorption in a material take place only when the incident photon has more energy than energy band gap of the material. Since TlBiS_2_ is a direct band gap material with a band gap of 1.10 eV, we notice the absorption to occur when the energy of the photon is around 1.08 eV, as shown in Figure 8. It is clearly seen in Figure 8 that there are absorption peaks at 1.32 eV, 1.93 eV, 2.45 eV, and 3.6 eV. The absorption coefficient of TlBiS_2_ has a maximum value when the photon energy is about 3.6 eV. For silicon, the absorption coefficient becomes appreciably different from zero after 2.5 eV, and it is still not very large up to 3 eV. This phenomenon can be attributed to the indirect band gap of silicon that leads to low absorption in the visible region. We observe that the absorption coefficient of TlBiS_2_ is superior to silicon in the visible region. This is due to the direct band gap at Г and F **k**-points that prevails in the TlBiS_2_.

In the case of hexagonal-Ba_3_BiN, the dielectric function is calculated at the BSE level (Figure 7b). Due to the narrow band gap, Ba_3_BiN can absorb photons mostly in the visible region. The HSE06 band gap is 1.29 eV, and it is direct. Therefore, Ba_3_BiN exhibits an absorption which rapidly increases after 1.26 eV. It can be observed that the absorption peaks of Ba_3_BiN are at 2.08 eV, 3.21 eV, and 3.5 eV (Figure 8). The absorption coefficient of Ba_3_BiN reaches its maximum when the photon energy is about 3.5 eV. From Figure 8, it can be noted that the optical absorption of Ba_3_BiN occurs in the visible region, with higher values compared to other materials considered. The reason behind the high optical absorption of Ba_3_BiN is due to the direct band gap of 1.29 eV. From Figure 8, it can be observed that absorption peaks of Ag_2_BaS_2_ are at 1.94 eV, 2.24 eV, 2.7 eV, and 3.6 eV. Ag_2_BaS_2_ seems to have a lower absorption coefficient than TlBiS_2_ and Ba_3_BiN in the visible region. However, Ag_2_BaS_2_ exhibits better optical absorption than silicon in the visible region. In the same Figure 8, we notice that absorption peaks of ZrSO start at 2.5 eV. The absorption peaks of ZrSO are also observed at 2.6 eV, 3 eV, and 3.59 eV. Among these four non-silicon materials, the absorption coefficient of ZrSO is smaller than those of the other three; this is due to the wideband gap of ZrSO.

## 4. Conclusions

We employed band gap calculations based on GGA on a pool of 1000 materials in order to identify twenty-seven possible candidates for photovoltaic applications. Among these candidates, four promising materials that had direct band gaps were chosen, and in-depth analysis was carried out to check the utility of these compounds for photovoltaic applications. We have presented a set of first-principle calculations employing the hybrid functional HSE06 and utilized to compute the electronic structures and effective masses of the four chosen materials, namely, TlBiS_2_, Ba_3_BiN, Ag_2_BaS_2_, and ZrSO. The BSE method was employed to calculate the optical properties. Our study provided rational insights into the electronic structure and optical properties of these four non-silicon materials. These four materials exhibit a direct band gap in the range of 1.10 eV to 2.60 eV.

The main advantage with TlBiS_2_, Ba_3_BiN, and Ag_2_BaS_2_ is that all three materials have direct band gaps and higher absorption coefficients than the widely-used photovoltaic material silicon in the visible region. Among these three materials, TlBiS_2_ and Ba_3_BiN have better optical properties than Ag_2_BaS_2_. ZrSO is the least preferable photovoltaic material because of the fact that its absorption properties are inferior to those of silicon. Nevertheless, we have shown that there is a significant difference in the GGA and HSE06 calculation for ZrSO. The phonon calculations revealed that TlBiS_2_, Ba_3_BiN, Ag_2_BaS_2_, and ZrSO are dynamically stable, as no imaginary frequencies were observed. Our elastic constant calculations illustrate that the compounds are mechanically stable. The calculated G/B values are greater than 0.5, confirming the brittle nature of these materials. Our detailed studies of the electronic, structural stability, mechanical stability and optical properties of these four materials show them to be potential candidates for photovoltaic application.

## Figures and Tables

**Table 1:** Calculated GGA and HSE06 band gap values, type of band gap for TlBiS_2_, Ba_3_BiN, Ag_2_BaS_2_, and ZrSO phases.

Compounds	GGA (eV)	HSE06 (eV)	Type of Band Gap
TlBiS_2_	0.505	1.10	direct
Ba_3_BiN	0.679	1.29	direct
Ag_2_BaS_2_	0.716	1.95	direct
ZrSO	0.891	2.60	direct

**Table 2:** The calculated effective mass of non-silicon compounds. m*_lh_, m*_hh_ and m*_e_ are the effective masses of light holes, heavy holes and electrons, respectively. m_e_ is the mass of the electron.

Serial No.	Plane Directions	Compound	m*_lh_ × m_e_	m*_hh_ × m_e_	m*_e_ × m_e_
1.	110	TlBiS_2_	0.182	0.224	0.154
2.	110	Ba_3_BiN	0.016	0.165	0.092
3.	110	Ag_2_BaS_2_	0.150	0.728	0.149
4.	110	ZrSO	0.308	0.482	0.361

**Table 3:** Computational details for the phonon calculation (supercell size, number of atoms), calculated zero-point energy (ZPE) and information on dynamical stability based on phonon density of states for TlBiS_2_, Ba_3_BiN, Ag_2_BaS_2_, and ZrSO phases.

Compounds	Supercell Size	Number of Atoms	ZPE (eV)	Dynamical Stability
TlBiS_2_	2 × 2 × 2	32	0.4045	stable
Ba_3_BiN	2 × 2 × 2	80	1.3050	stable
Ag_2_BaS_2_	2 × 2 × 2	40	0.1093	stable
ZrSO	2 × 2 × 2	48	1.2100	stable

**Table 4:** The calculated single-crystal elastic constants *C_ij_* (in GPa), bulk modulus B (in GPa), shear modulus G (in GPa), Poisson’s ratio (σ), Young’s modulus E (in GPa), compressibility (GPa^−1^), Ductility for TlBiS_2_, Ba_3_BiN, Ag_2_BaS_2_ and ZrSO phases. Subscript V illustrates the Voigt bound, R indicates the Reuss bound and VRH indicates the Hill average.

Properties	TlBiS_2_	Ba_3_BiN	Ag_2_BaS_2_	ZrSO
*C_ij_*	C_11_ = 63.47 C_12_ = 34.68 C_13_ = 27.49 C_14_ = 0.608 C_33_ = 88.90 C_44_ = 28.90	C_11_ = 56.604 C_12_ = 14.1 C_13_ = 7.947 C_33_ = 75.35 C_44_ = 21.20	C_11_ = 84.61 C_12_ = 31.538 C_13_ = 34.611 C_14_ = 0.0732 C_33_ = 89.994 C_44_ = 26.642 C_66_ = 14.590	C_11_ = 293.04 C_12_ = 133.66 C_13_ = 118.96 C_33_ = 303.06 C_44_ = 100.64 C_66_ = 91.94
**B_V_**	44.85	27.762	51.59	181.407
**B_R_**	44.46	27.5620	51.28	181.39
**B_VRH_**	44.65	27.662	51.34	181.403
**G_V_**	54.99	47.7264	53.45	194.92
**G_R_**	21.08	5.70729	19.24	90.88
**G_VRH_**	38.04	26.716	36.35	142.90
**Ductility**	0.852	0.96	0.708	0.7877
**σ**	0.168	0.1346	0.213	0.188
**E**	89	61	29	339
**compressibility**	0.022	0.04	0.02	0.0055

## Supplementary Materials

The following are available online at http://www.mdpi.com/1996-1944/11/10/2006/s1, Table S1: Calculated GGA band gap values for 30 compounds with lattice parameters; Table S2: Calculated structural parameters and atomic positions of TlBiS_2_, Ba_3_BiN, Ag_2_BaS_2_, and ZrSO; Figure S1: Calculated total energy as a function of unit cell volume for cubic- and tetragonal-ZrSO; Figure S2: Crystal structures for (a) cubic-ZrSO; (b) tetragonal-ZrSO. The legends for the different kinds of atoms shown in the illustration; Figure S3: Total and site projected density of states of Ba_3_BiN. The Fermi level is set to zero and marked by a vertical dotted line; Figure S4: Total and site projected density of states of Ag_2_BaS_2_. The Fermi level is set to zero and marked by a vertical dotted line; Figure S5: Total and site projected density of states of ZrSO. The Fermi level is set to zero and marked by a vertical dotted line.
