# Supplementary material for: Properties of Novel Non-Silicon Materials for Photovoltaic Applications: A First-Principle Insight
Source: Materials (Basel). 2018 Oct 17;11(10):2006. doi: 10.3390/ma11102006 (PMC6212800; doi:10.3390/ma11102006)
Supplement: Supplementary file 1 [file materials-11-02006-s001.pdf]

materials

MDPI

Article

Properties of Novel Non-Silicon Materials for Photovoltaic Applications: A First-Principle Insight

Murugesan Rasukkannu [1,*], Dhayalan Velauthapillai [1], Federico Bianchini [2] and Ponniah Vajeeston [2]

[1] Department of Computing, Mathematics, and Physics, Western Norway University of Applied Sciences, Inndalsveien 28, 5063, Bergen, Norway; vdh@hvl.no
[2] Department of Chemistry, Center for Materials Science and Nanotechnology, University of Oslo, Box 1033 Blindern N-0315, Oslo, Norway; federico.bianchini@smn.uio.no (F.B.); ponniah.vajeeston@kjemi.uio.no (P.V.)
* Correspondence: rmu@hvl.no Tel.: +47-5558-7786

Received: 18 September 2018; Accepted: 11 October 2018; Published: 17 October 2018

Table S1. Calculated GGA band gap values for 30 compounds with lattice parameters.

S.No	Chemical formula	Space group	Pearson symbol	Lattice Parameter(computed)			Energy gap (in eV)	Type of band gap
				a	b	c		
1	$TlBiS_2$	R-3m(166)	hR4	7.817			0.5055	Direct
2	$BaGe_2$	*Pnma* (62)	oP24	6.860	9.196	11.678	0.5235	Indirect
3	Gd_2S_3	*Pnma* (62)	oP20	3.930	10.580	10.790	0.526	Direct
4	$GaTlTe_2$	*I4/mcm* (140)	tI16	7.020			0.5618	Indirect
5	GeP	*C2/m* (12)	mS24	7.990		9.350	0.5719	Direct
6	Ca_2CuFeO_3S	*P4/nmm* (129)	tP16	3.88		14.94	0.6339	Indirect
7	$Fe_2Ga_2S_5$	*P*-3*m*1 (164)	hP9	3.690		15.570	0.6558	Indirect
8	$Ca_2Fe_2O_5$	*Pnma* (62)	oP36	5.538	5.6589	14.885	0.6614	Direct
9	Ba_3BiN	$P6_3/mmc$(194)	hP10	7.770		6.805	0.6705	Direct
10	Cu_2GeZnS_4	*I*-42*m* (121)	tI16	6.552			0.6729	Direct
11	$CdCu_2GeS_4$	$Pmn2_1$ (31)	oP16	6.359	6.627	7.779	0.6863	Direct
12	$CdGeP_2$	*I*-42*d* (122)	tI16	6.838			0.6942	Direct
13	$ZnSnP_2$	*I*-42*d* (122)	tI16	7.0146			0.6984	Direct
14	$CdMn_2O_4$	$I4_1/amd$ (141)	tI28	6.5092			0.7017	Indirect
15	Ag_2BaS_2	*P*-3*m*1 (164)	hP9	4.4251		7.2810	0.7161	Direct
16	$CuKZrS_3$	*Cmcm* (63)	oS24	7.3557		9.8515	0.7388	Direct
17	$CuFeO_3SSr_2$	*P4/nmm* (129)	tP16	3.9557		15.7502	0.7516	Indirect
18	$KMnNaO_2$	*Cccm* (66)	oS40	6.8241		7.1011	0.7749	Direct
19	Cu_2GeS_4Zn	$Pmn2_1$ (31)	oP16	6.2223	6.5706	7.4744	0.7798	Direct
20	Fe_2MnO_4	*Fd*-3*m* (227)	cF56	6.1012			0.7842	Indirect
21	MnO_3Sr	P63/mmc(194)	hP20	5.5350		9.2802	0.8325	Indirect
22	Cu_2GeMnS_4	$Pmn2_1$ (31)	oP16	6.2986	6.5754	7.6952	0.886	Indirect
23	ZrSO	*P4/nmm* (129)	tP6	3.6280		6.4154	0.8964	Direct
24	$Mn_2Na_{14}O_9$	*P*-3 (147)	hP25	6.7098		9.4004	0.9245	Indirect
25	$FeGeO_3$	*C2/c* (15)	mS40	6.7799		5.2895	0.9459	Indirect
26	$K_3Ni_2O_4$	*Cmcm* (63)	oS36	5.5013		10.6834	1.0845	Indirect
27	$MnNaO_2$	*Pmmn* (59)	oP8	5.8575		6.7005	1.1331	Indirect

The initial structural parameters of a thousand compounds were directly taken from the ICSD database[1], and then GGA band gap for thousand non-silicon compounds were calculated in our DFTB database[2]. These are multinary compounds including conductors, semiconductors, and insulators. Among these thousand non-silicon compounds we considered twenty-seven of them with GGA band gap values in the range of 0.5–1.1 eV (Table S1). Among these twenty-seven compounds, we identified fourteen compounds as direct band gap semiconductors and thirteen as indirect band gap semiconductors. We carried out a study on both electronic and optical properties of twenty-seven semiconductors (both direct and indirect). Our study on the optical properties of the semiconductor

materials showed that four direct band gaps among the twenty-seven materials had higher absorption coefficients in the visible region. Due to the space constraint, the optical properties of all the twenty-seven semiconductors are not presented in the supporting information part

Table S2. Calculated structural parameters and atomic positions of $TlBiS_2$, Ba_3BiN, Ag_2BaS_2, and ZrSO.

**Phase**	**Lattice parameter** *a*	*b*	*c*	*α(deg)*	*β(deg)*	*γ(deg)*	***Atomic positions***
$TlBiS_2$ -R-3m ;166	7.817(7.711[a])			30.83	30.83	30.83	[a]Bi(3a): 0.000 0.000 0.000<br>[a]Tl(9d): ½ ½ ½<br>[a]S(36i) : 0.237 0.237 0.237
Ba_3BiN-$P6_3$/mmc;194	7.770 (7.6128[b])		6.805 (6.6805[b])	90	90	120	[b]Ba(6h): 0.1605 -0.1605 ¼<br>[b]Bi(2d): 1/3 2/3 ¾<br>[b]N(2a): 0.000 0.000 0.000
Ag_2BaS_2- *P*-3*m*1 ;164	4.4251(4.3861[c])		7.2810(7.1942[c])	90	90	120	[c]S(2d) : 1/3 2/3 0.25296<br>[c]Ba(1a) : 0.000 0.000 0.000<br>[c]Ag(2d) : 1/3 2/3 0.62252
ZrSO-P4/nmm; 129	3.6280		6.4154	90	90	90	[d]Zr(*2c*): 0, ½, 0.1950<br>[d]S(*2c*): 0, ½, 0.6330<br>[d]O(*2a*): 0, 0, 0
ZrSO-$P2_13$; 198	5.6960						[e]Zr(*4a*): 0.071, 0.071, 0.071<br>[e]S(*4a*): 0.3335, 0.3335, 0.3335<br>[e]O(*4a*): 0.6535, 0.6535, 0.6535

[a]Reference [3]; [b]Reference [4]; [c]Reference [5]; [d]Reference [6]; [e]Reference [7].

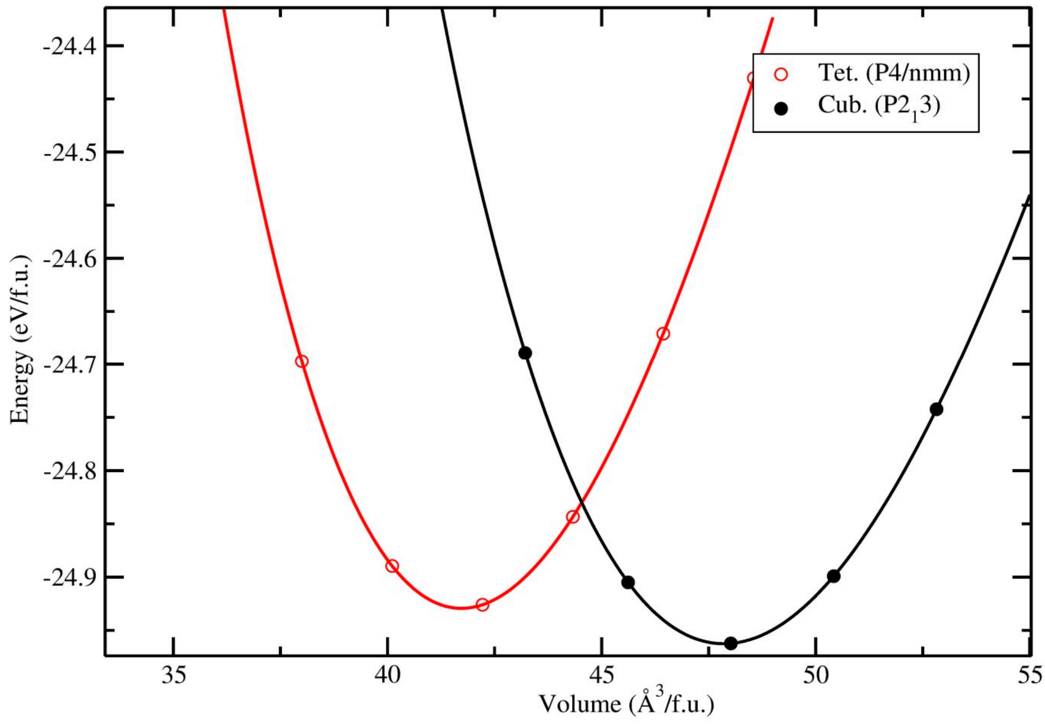

Figure S1. Calculated total energy as a function of unit cell volume for cubic- and tetragonal-ZrSO.

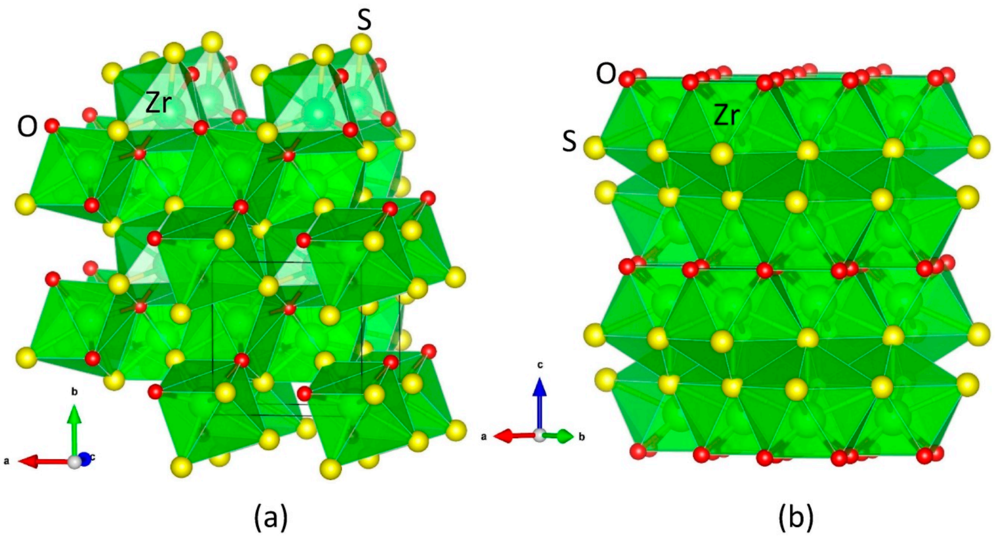

Figure S2. Crystal structures for (**a**) cubic-ZrSO; (**b**) tetragonal-ZrSO. The legends for the different kinds of atoms shown in the illustration.

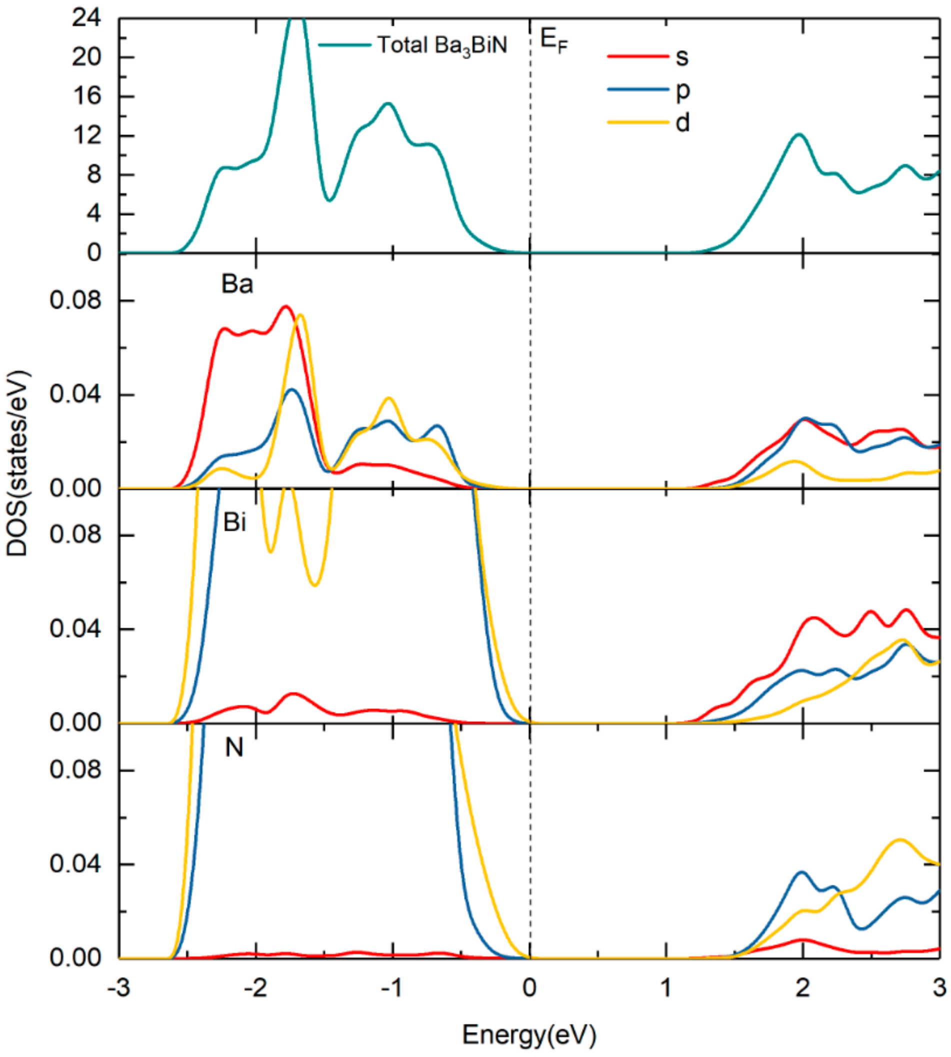

Figure S3. Total and site projected density of states of Ba_3BiN. The Fermi level is set to zero and marked by a vertical dotted line.

The total and site projected density of states of Ba_3BiN, Ag_2BaS_2, and ZrSO are presented in Figure S3, S4 and S5 respectively. From **Error! Reference source not found.**, we observe that the valence band derived from Bi-p and hybridized Ni-d states and conduction bands are mainly composed of Bi-*s* and Ba-*s* states. From Figure S4, we observe that the valence band maximum is derived from S-*p* states and the conduction band derived from Ag-*s* states. In the case of ZrSO, we observe that the valence bands derived from S-*p* states, and conduction bands derived from O-*s* states as shown in Figure S5.

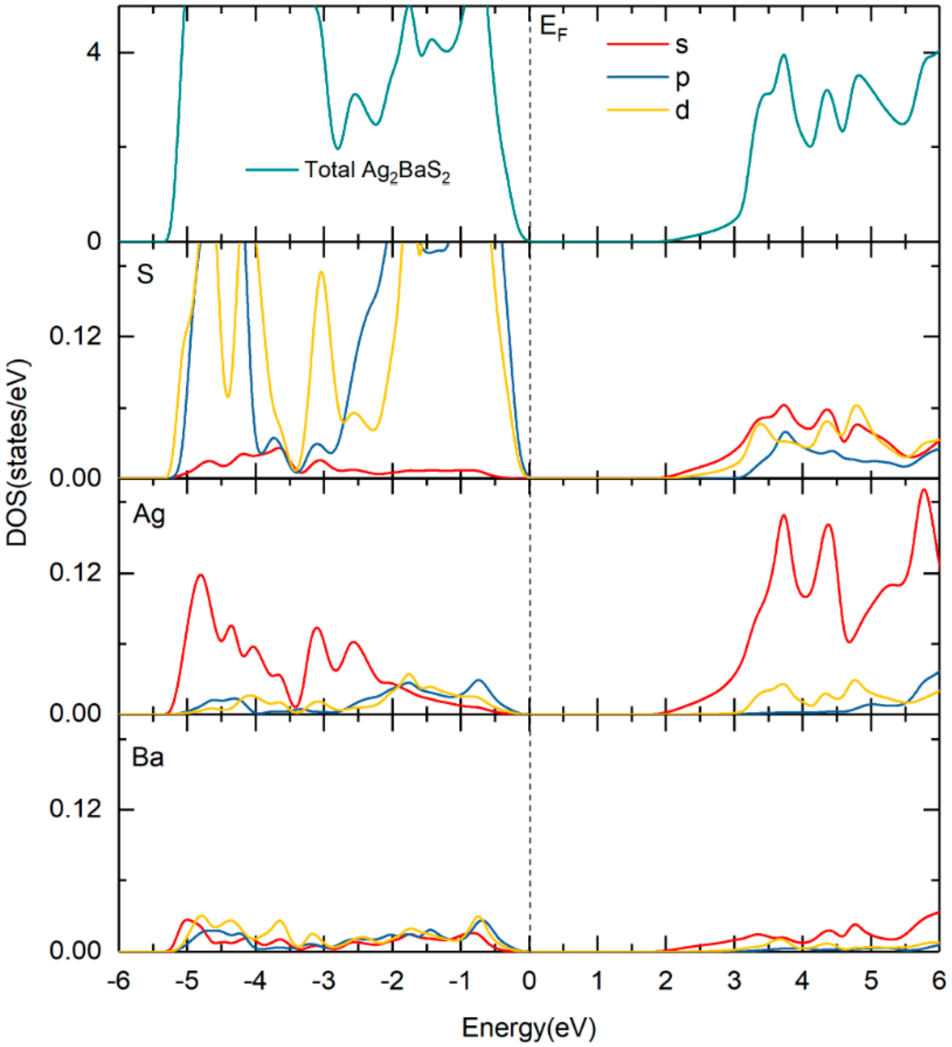

Figure S4. Total and site projected density of states of Ag_2BaS_2. The Fermi level is set to zero and marked by a vertical dotted line.

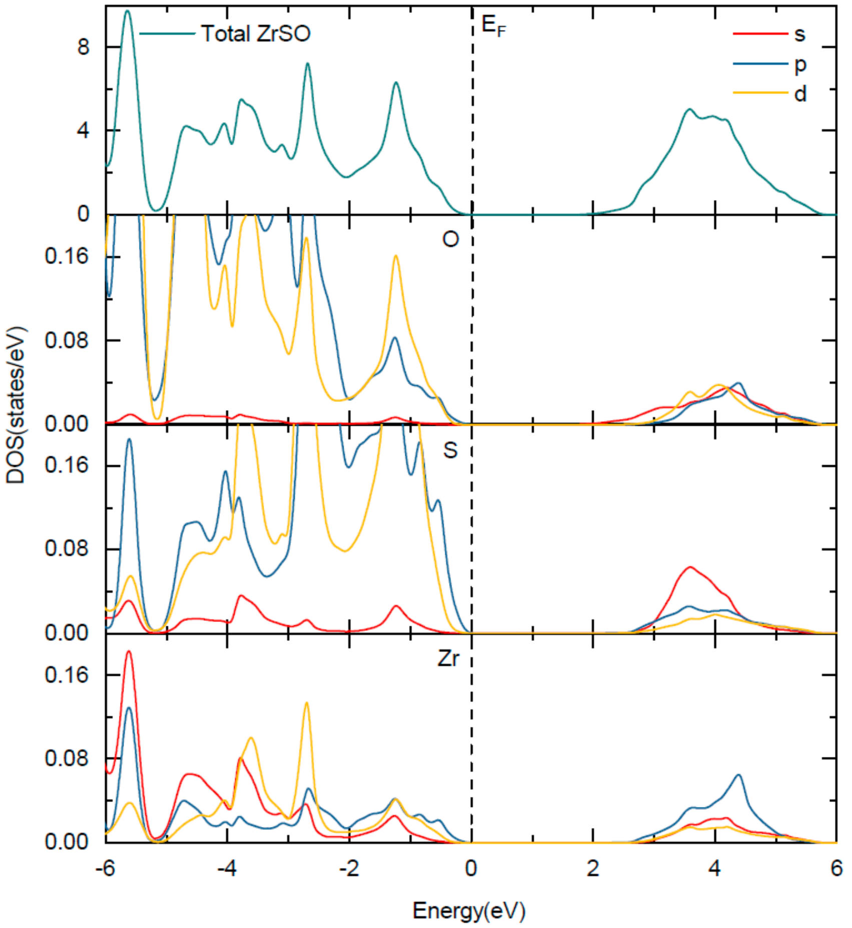

Figure S5. Total and site projected density of states of ZrSO. The Fermi level is set to zero and marked by a vertical dotted line.

References

1. Hellenbrandt, M. The inorganic crystal structure database (ICSD)—present and future. *Crystallogr. Rev.* **2004**, *10*, 17–22.
2. Ponniah, V. *Density Functional Theory Based Database (DFTBD)*. University of Oslo, Norway, 2013.
3. Özer, M.; Paraskevopoulos, K.; Anagnostopoulos, A.; Kokou, S.; Polychroniadis, E. Large single-crystal growth and characterization of the narrow-gap semiconductor. *Semicond. Sci. Technol.* **1996**, *11*, 1405.
4. Gäbler, F.; Kirchner, M.; Schnelle, W.; Schwarz, U.; Schmitt, M.; Rosner, H.; Niewa, R. (Sr3N) E and (Ba3N) E (E= Sb, Bi): synthesis, crystal structures, and physical properties. *Zeitschrift für anorganische und allgemeine Chemie* **2004**, *630*, 2292–2298.
5. Bronger, W.; Lenders, B.; Huster, J. BaAg2S2, ein Thioargentat im CaAl2Si2-Strukturtyp. *Zeitschrift für anorganische und allgemeine Chemie* **1997**, *623*, 1357–1360.
6. Jellinek, F.; Songstad, J.; Viljanto, J.; Seppälä, P.; Theander, O.; Flood, H. A Tetragonal Form of Zirconium Oxide Sulfide, ZrOS. *Acta Chem. Scand.* **1962**, *16*, 791–792, doi:10.3891/acta.chem.scand.16-0791.
7. McCullough, J.D.; Brewer, L.; Bromley, L. The crystal structure of zirconium oxysulfide, ZrOS. *Acta Crystallogr.* **1948**, *1*, 287–289.

© 2018 by the authors; licensee MDPI, Basel, Switzerland. This article is an open access article distributed under the terms and conditions of the Creative Commons Attribution (CC BY) license (http://creativecommons.org/licenses/by/4.0/).
